# Association between COVID-19 testing uptake and mental disorders among adults in US post-secondary education, 2020–2021

**DOI:** 10.1192/bjo.2022.580

**Published:** 2022-09-27

**Authors:** Yusen Zhai, Xue Du

**Affiliations:** Department of Human Studies, The University of Alabama at Birmingham, Birmingham, Alabama, USA; Department of Food Science, The Pennsylvania State University, University Park, Pennsylvania, USA. The work described in the paper was carried out at the University of Alabama at Birmingham, USA

**Keywords:** Depressive disorders, anxiety disorders, COVID-19, suicide, psychosocial interventions

## Abstract

Fear and uncertainty have worsened mental health outcomes during the COVID-19 pandemic. COVID-19 testing is essential yet underutilised, and many people may experience difficulties accessing testing if the US federal government fails to sustain the testing capacity. To date, limited evidence exists about the role of COVID-19 testing in mental health. We examined the associations of COVID-19 testing uptake with certain mental disorders, through a nationally representative cohort of adults in US post-secondary education (*N* = 65 360). Adults with test-confirmed COVID-19 were at significantly lower risk than those with unconfirmed COVID-19 for severe depression, severe anxiety, eating disorders, and suicidal ideation. Findings suggest another potential benefit of public health efforts to encourage COVID-19 testing, namely promoting mental health.

Testing is an effective yet underutilised means of managing the transmission of COVID-19.^1,2^ In the USA, it has been difficult to get COVID-19 tests for much of the pandemic,^2^ and many individuals may again experience difficulties obtaining testing after shortages if the US federal government can no longer sustain COVID-19 testing capacity without sufficient funding from Congress.^3^ Fear and uncertainty fuelled by COVID-19 among public and healthcare workers have worsened mental health outcomes, overwhelming and exhausting the healthcare system.^4–6^ Despite the current growing body of knowledge of testing and neuropsychiatric symptoms associated with COVID-19,^7,8^ limited evidence exists about the role of COVID-19 testing in mental health outcomes; namely, whether individuals with test-confirmed COVID-19 are less susceptible to certain mental disorders compared with those with unconfirmed COVID-19. In the current study, we hypothesised that COVID-19 testing uptake was significantly associated with severe depression, severe anxiety, eating disorders, and suicidal ideation.

## Method

We analysed data from the Healthy Minds Network, which surveyed a random sample of individuals 18 years and older from 140 US colleges and universities (including non-degree programmes) from 9 September 2020 to 24 May 2021. Sample weights were used to adjust non-response based on institutional data on biological sex, race/ethnicity, academic level, and grade point average. Primary outcomes included severe depression symptoms (≥15 on the Patient Health Questionnaire-9), severe anxiety symptoms (≥15 on the Generalised Anxiety Disorder-7 questionnaire), eating disorders (≥3 on the Sick, Control, One, Fat, Food (SCOFF) questionnaire), and suicidal ideation (‘yes’ on ‘In the past year, did you ever seriously think about attempting suicide’). We categorised participants into four groups based on their responses: group 1 (reference group), COVID-19 diagnosis confirmed by testing; group 2, physician-diagnosed COVID-19 without testing; group 3, experienced symptoms consistent with COVID-19 without testing; and group 4, no COVID-19 symptoms. We performed sample-weighted multivariable logistic regression analysis to compute adjusted odds ratios (aORs) and 95% confidence intervals, adjusting for covariates. Based on previous studies,^9,10^ covariates included: (a) demographic variables, i.e. age, in years; sex (i.e. female, male, other (intersex)); gender (e.g. woman, man, trans); race/ethnicity (e.g. Black/African American, Asian, Latinx (individuals of Latin American origin or descent), White); disability; international (i.e. international student on a temporary, non-immigrant US visa); relationship status (e.g. single, in a relationship, married); residence (e.g. on-campus, off-campus); and (b) plausible risk factors for mental disorders, i.e. chronic disease (e.g. diabetes, hypertension), smoking and vaping status, and history of diagnosed mental disorder (e.g. clinical depression, anxiety, psychosis). A two-sided *P* < 0.05 was considered statistically significant.

The authors assert that all procedures contributing to this work comply with the ethical standards of the relevant national and institutional committees on human experimentation and with the Helsinki Declaration of 1975, as revised in 2008. All procedures involving human participants were approved by the institutional review board (IRB-300008474) of the University of Alabama at Birmingham. Written informed consent was obtained from all participants.

## Results

**Fig. 1:**
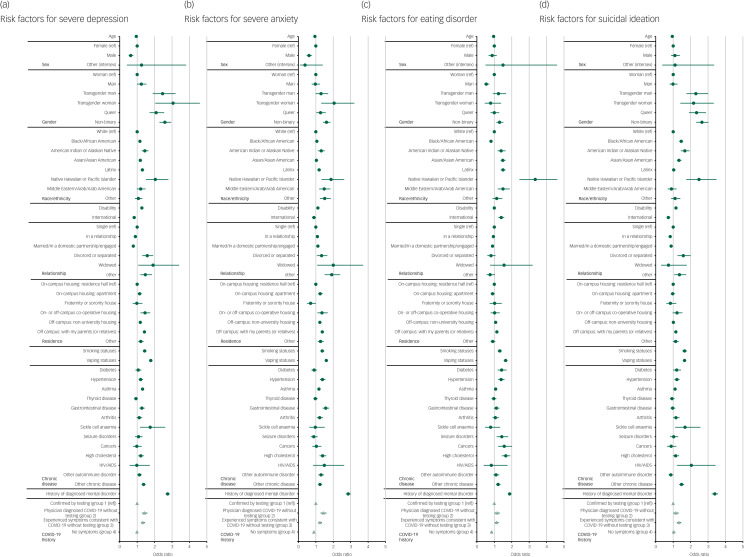
Associations of mental disorders with demographics, chronic diseases, and COVID-19 testing uptake among adults in post-secondary education in the USA, 2020–2021. ref, reference; International, international student on a temporary, non-immigrant US visa.

The current study included 65 360 participants (mean age 24.2 years, s.d. = 8.2 years; 57.7% women). The supplementary Table available at https://doi.org/10.1192/bjo.2022.580 presents participants’ demographics. The prevalence of severe depression, severe anxiety, eating disorders, and suicidal ideation were 21.2%, 16.4%, 11.7%, and 13.5% respectively. Fig. 1 presents the risk factors for these primary outcomes.

Participants with physician-diagnosed COVID-19 but unconfirmed by testing (group 2) (aOR = 1.42, 95% CI 1.27–1.59, *P* < 0.001) and those with suspected COVID-19 without testing (group 3) (aOR = 1.33, 95% CI 1.23–1.45, *P* < 0.001) were significantly more likely to suffer severe depression compared with those with test-confirmed COVID-19 (group 1).

Participants with physician-diagnosed COVID-19 but unconfirmed by testing (group 2) (aOR = 1.43, 95% CI 1.27–1.61, *P* < 0.001) and those with suspected COVID-19 without testing (group 3) (aOR = 1.24, 95% CI 1.14–1.35, *P* < 0.001) were significantly more likely to suffer severe anxiety compared with those with test-confirmed COVID-19 (group 1) .

Participants with physician-diagnosed COVID-19 but unconfirmed by testing (group 2) (aOR = 1.16, 95% CI 1.01–1.32, *P* = 0.030) and those with suspected COVID-19 without testing (group 3) (aOR = 1.13, 95% CI 1.03–1.25, *P* = 0.013) were significantly more likely to suffer eating disorders compared with those with test-confirmed COVID-19 (group 1).

Participants with physician-diagnosed COVID-19 but unconfirmed by testing (group 2) (aOR = 1.15, 95% CI 1.01–1.32, *P* = 0.036) and those with suspected COVID-19 without testing (group 3) (aOR = 1.33, 95% CI 1.21–1.46, *P* < 0.001) were significantly more likely to have suicidal ideation compared with those with test-confirmed COVID-19 (group 1).

Significant differences were found in severe anxiety and eating disorders but not in severe depression or suicidal ideation between participants without COVID-19 symptoms (group 4) and those with test-confirmed COVID-19 (group 1).

## Discussion

To our current knowledge, this is the first national study to examine associations between COVID-19 testing uptake and mental disorders. These findings support our hypothesis that adults with test-confirmed COVID-19 were at significantly lower risk for severe depression, severe anxiety, eating disorders and suicidal ideation compared with those with suspected or physician-diagnosed COVID-19 unconfirmed by testing. These findings corroborate previous research indicating that individuals who suspected that they had COVID-19 but lacked confirmatory testing were more susceptible to exacerbated mental health problems due to fear of infection.^11,12^ These findings suggest that testing may help adults with COVID-19-like symptoms to reduce the risk for mental distress associated with the fear and uncertainty of infection and consequent restrictions.

Additionally, we found no significant difference in risk for severe depression and suicidal ideation between adults with test-confirmed COVID-19 and those without COVID-19 symptoms. Considering that studies suggest COVID-19 infection increases the risk for neuropsychiatric symptoms,^7,8^ we conclude that testing may help attenuate the risk for severe depression and suicidal ideation exacerbated by COVID-19 symptoms. Significant differences found in severe anxiety and eating disorders between these two groups might be due to COVID-19 infection rather than testing.^7,8^ Some people with a history of COVID-19 infection might suffer long-term physical and mental sequelae of the disease (‘long COVID’) and adverse effects of medications treating infections, which could fuel excessive worry and dysregulate eating behaviours/patterns.^13,14^ These findings suggest that mental health professionals should be ready to address individuals’ mental health problems that develop after COVID-19 illness. To prevent mental disorders associated with severe infection, mental health professionals should also encourage preventive measures (e.g. testing, vaccination) and promote people's health literacy.^15^

This study has limitations. First, the generalisability may be limited because participants represented adults enrolled in post-secondary education. Although our sample showed a wide age range (18–65+ years), the majority were young adults. Older adults may have different levels of fear and tolerance of uncertainty regarding COVID-19. Future studies might examine the role of testing uptake in mental health across different populations/settings. Moreover, causal inferences should be interpreted with caution in this study as cross-sectional data restricted our ability to determine the temporal order between testing uptake and mental disorders that were measured simultaneously. Longitudinal studies are therefore needed to help assess the temporal links. Despite these limitations, this study provides empirical evidence for the associations between COVID-19 testing uptake and mental disorders through a large representative cohort, controlled for covariates to reduce possible confounding effects.

This study underscores the vital role of mental health professionals in public health, providing preliminary support for another potential benefit of public health efforts to encourage COVID-19 preventive measures,^15^ namely promoting mental health. When coupled with other public health measures, timely COVID-19 testing can reduce the transmission of SARS-CoV-2 extensively and can also reduce the risk for severe depression, severe anxiety, eating disorders and suicidal ideation. Our findings suggest that encouraging COVID-19 testing and making testing more accessible will lessen the strain of the pandemic on our healthcare systems and public mental health, particularly during any resurgence of COVID-19 infections.

## Data Availability

The data that support the findings of this study are openly available on request from the Healthy Minds Network at https://healthymindsnetwork.org.

## Supplementary material

For supplementary material accompanying this paper visit https://doi.org/10.1192/bjo.2022.580. click here to view supplementary material
