# Supplementary material for: Association between COVID-19 testing uptake and mental disorders among adults in US post-secondary education, 2020–2021
Source: BJPsych Open. 2022 Sep 27;8(5):e171. doi: 10.1192/bjo.2022.580 (PMC9530375; doi:10.1192/bjo.2022.580)
Supplement: Supplementary file 1 [file S2056472422005804sup001.docx]

| Supplementary Table. Characteristics of 65360 Participants and Results of Multivariable Logistic Regression Analyses of Risk Factors | | | | | | | | | |
| --- | --- | --- | --- | --- | --- | --- | --- | --- | --- |
|  |  | **Severe Depression** | | **Severe Anxiety** | | **Eating Disorders** | | **Suicidal Ideation** | |
| **Variable** | **No. (weighted %)** | **aOR (95% CI)** | ***P* value** | **aOR (95% CI)** | ***P* value** | **aOR (95% CI)** | ***P* value** | **aOR (95% CI)** | ***P* value** |
| **Age** |  | **0.95 (0.95-0.95)** | <.001 | **0.95 (0.95-0.96)** | <.001 | **0.96 (0.96-0.97)** | <.001 | **0.95 (0.94-0.95)** | <.001 |
| **Sex** |  |  |  |  |  |  |  |  |  |
| Female | 39478(60.4) | 1 (reference) | NA | 1 (reference) | NA | 1 (reference) | NA | 1 (reference) | NA |
| Male | 25791(39.5) | **0.63 (0.51-0.78)** | <.001 | **0.61 (0.48-0.77)** | <.001 | 0.88 (0.67-1.14) | .337 | 1.10 (0.88-1.38) | .397 |
| Other (intersex) | 19(.03) | 1.25 (0.41-3.81) | .698 | 0.38 (0.10-1.40) | .146 | 1.50 (0.49-4.60) | .477 | 1.11 (0.37-3.35) | .851 |
| **Gender** |  |  |  |  |  |  |  |  |  |
| Woman | 37688(57.7) | 1 (reference) | NA | 1 (reference) | NA | 1 (reference) | NA | 1 (reference) | NA |
| Man | 25132(38.5) | 1.24 (0.99-1.53) | .056 | 0.97 (0.76-1.24) | .826 | **0.54 (0.41-0.71)** | <.001 | 0.99 (0.79-1.25) | .956 |
| Transgender Man | 263(.4) | **2.46 (1.90-3.20)** | <.001 | 1.30 (0.99-1.71) | .060 | 1.24 (0.91-1.68) | .171 | **2.30 (1.76-3.01)** | <.001 |
| Transgender Woman | 148(.2) | **3.06 (2.04-4.60)** | <.001 | **2.05 (1.31-3.21)** | .002 | 0.79 (0.45-1.39) | .417 | **2.17 (1.41-3.33)** | <.001 |
| Queer | 506(.8) | **2.09 (1.71-2.55)** | <.001 | **1.27 (1.03-1.57)** | .028 | 1.00 (0.78-1.28) | .983 | **2.34 (1.90-2.88)** | <.001 |
| Non-binary | 1337(2.0) | **2.59 (2.28-2.95)** | <.001 | **1.61 (1.41-1.84)** | <.001 | **1.30 (1.12-1.52)** | .001 | **2.65 (2.32-3.03)** | <.001 |
| **Race** |  |  |  |  |  |  |  |  |  |
| White | 43312(66.3) | 1 (reference) | NA | 1 (reference) | NA | 1 (reference) | NA | 1 (reference) | NA |
| Black/African American | 7963(12.2) | **1.16 (1.09-1.25)** | <.001 | 1.06 (0.98-1.14) | .125 | **0.81 (0.74-0.89)** | <.001 | **1.46 (1.35-1.57)** | <.001 |
| American Indian or Alaskan Native | 1291(2.0) | **1.44 (1.26-1.65)** | <.001 | **1.31 (1.13-1.51)** | <.001 | **1.39 (1.19-1.64)** | <.001 | **1.67 (1.44-1.94)** | <.001 |
| Asian/Asian American | 5591(8.6) | **1.18 (1.09-1.28)** | <.001 | 1.03 (0.94-1.13) | .549 | **1.49 (1.36-1.64)** | <.001 | **1.33 (1.21-1.47)** | <.001 |
| Latinx | 5397(8.3) | **1.30 (1.21-1.40)** | <.001 | **1.19 (1.09-1.29)** | <.001 | **1.50 (1.37-1.64)** | <.001 | 1.03 (0.94-1.13) | .544 |
| Native Hawaiian or Pacific Islander | 224(.3) | **2.04 (1.50-2.79)** | <.001 | **1.87 (1.33-2.63)** | <.001 | **3.35 (2.44-4.62)** | <.001 | **2.48 (1.75-3.49)** | <.001 |
| Middle Eastern/Arab/Arab American | 627(1.0) | 1.20 (0.98-1.48) | .081 | **1.48 (1.19-1.84)** | <.001 | **1.50 (1.19-1.89)** | .001 | 0.90 (0.68-1.19) | .449 |
| Other | 833(1.3) | 1.07 (0.87-1.30) | .542 | **1.51 (1.23-1.86)** | <.001 | 1.14 (0.90-1.46) | .280 | 1.11 (0.88-1.41) | .380 |
| **Disability** | 5514(8.4) | **1.27 (1.19-1.36)** | <.001 | **1.12 (1.04-1.20)** | .002 | 1.00 (0.92-1.09) | .966 | **1.16 (1.07-1.25)** | <.001 |
| **International** | 3264(5.0) | **0.83 (0.75-0.93)** | .002 | 0.89 (0.78-1.01) | .071 | **1.40 (1.24-1.57)** | <.001 | **0.72 (0.62-0.83)** | <.001 |
| **Relationship** |  |  |  |  |  |  |  |  |  |
| Single | 32859(50.3) | 1 (reference) | NA | 1 (reference) | NA | 1 (reference) | NA | 1 (reference) | NA |
| In a relationship | 21763(33.3) | **0.90 (0.86-0.94)** | <.001 | **1.09 (1.04-1.15)** | <.001 | 0.95 (0.90-1.01) | .088 | **0.83 (0.79-0.87)** | <.001 |
| Married/in a domestic partnership/engaged | 9346(14.3) | **0.78 (0.72-0.84)** | <.001 | **1.12 (1.02-1.21)** | .012 | **0.90 (0.81-0.99)** | .029 | **0.88 (0.80-0.97)** | .008 |
| Divorced or separated | 781(1.2) | **1.58 (1.29-1.92)** | <.001 | **1.33 (1.06-1.66)** | .012 | 0.81 (0.60-1.07) | .141 | **1.58 (1.25-2.00)** | <.001 |
| Widowed | 74(.1) | **1.91 (1.06-3.41)** | .030 | **2.00 (1.08-3.72)** | .028 | 1.56 (0.76-3.20) | .222 | 0.73 (0.30-1.78) | .489 |
| Other | 463(.7) | **1.47 (1.18-1.85)** | .001 | **1.91 (1.51-2.40)** | <.001 | 0.76 (0.55-1.04) | .083 | **1.36 (1.06-1.75)** | .017 |
| **Residence** |  |  |  |  |  |  |  |  |  |
| On-campus housing: residence hall | 14217(21.8) | 1 (reference) | NA | 1 (reference) | NA | 1 (reference) | NA | 1 (reference) | NA |
| On-campus housing: apartment | 3111(4.8) | **1.14 (1.03-1.26)** | .012 | **1.25 (1.12-1.40)** | <.001 | 0.90 (0.79-1.03) | .123 | 0.97 (0.86-1.10) | .630 |
| Fraternity or sorority house | 332(.5) | 0.98 (0.74-1.30) | .893 | **0.70 (0.50-1.00)** | .047 | 1.02 (0.73-1.43) | .903 | 0.84 (0.61-1.18) | .321 |
| On- or off-campus co-operative housing | 687(1.1) | **1.45 (1.19-1.75)** | <.001 | **1.35 (1.09-1.68)** | .006 | 1.02 (0.79-1.31) | .868 | 1.22 (0.97-1.53) | .094 |
| Off-campus: non-university housing | 25191(38.5) | **1.18 (1.11-1.25)** | <.001 | **1.23 (1.15-1.31)** | <.001 | **1.08 (1.01-1.16)** | .035 | 1.01 (0.94-1.09) | .713 |
| Off campus: with my parents (or relatives) | 18986(29.0) | **1.42 (1.34-1.50)** | <.001 | **1.37 (1.29-1.47)** | <.001 | **1.15 (1.07-1.24)** | <.001 | **1.15 (1.08-1.23)** | <.001 |
| Other | 2779(4.3) | **1.21 (1.06-1.37)** | .004 | **1.27 (1.11-1.45)** | .001 | 0.91 (0.78-1.06) | .238 | 1.14 (0.98-1.32) | .085 |
| **Smoking statues** | 4319(6.6) | **1.43 (1.32-1.54)** | <.001 | **1.37 (1.26-1.49)** | <.001 | **1.31 (1.19-1.44)** | <.001 | **1.65 (1.52-1.80)** | <.001 |
| **Vaping statues** | 9176(14.0) | **1.78 (1.68-1.88)** | <.001 | **1.60 (1.51-1.70)** | <.001 | **1.65 (1.54-1.76)** | <.001 | **1.65 (1.55-1.75)** | <.001 |
| **Chronic disease** |  |  |  |  |  |  |  |  |  |
| Diabetes | 1214(1.9) | 1.06 (0.90-1.24) | .522 | 0.90 (0.75-1.07) | .236 | **1.42 (1.19-1.71)** | <.001 | 1.20 (0.99-1.45) | .060 |
| Hypertension | 2955(4.5) | **1.20 (1.08-1.34)** | .001 | **1.38 (1.23-1.55)** | <.001 | **1.39 (1.22-1.59)** | <.001 | **1.21 (1.07-1.37)** | .003 |
| Asthma | 9530(14.6) | **1.31 (1.24-1.38)** | <.001 | **1.18 (1.11-1.25)** | <.001 | **1.08 (1.01-1.15)** | .030 | **1.11 (1.04-1.19)** | .001 |
| Thyroid disease | 2067(3.2) | 0.93 (0.82-1.04) | .210 | 0.97 (0.86-1.10) | .656 | 0.97 (0.84-1.12) | .678 | 0.93 (0.81-1.08) | .348 |
| Gastrointestinal disease | 1631(2.5) | **1.27 (1.13-1.43)** | <.001 | **1.57 (1.39-1.77)** | <.001 | 1.12 (0.97-1.29) | .134 | 0.97 (0.84-1.12) | .675 |
| Arthritis | 1500(2.3) | 1.12 (0.97-1.29) | .113 | **1.22 (1.06-1.41)** | .006 | 1.06 (0.90-1.26) | .478 | 1.16 (0.99-1.37) | .068 |
| Sickle cell anemia | 138(.2) | **1.75 (1.17-2.61)** | .006 | 0.97 (0.62-1.51) | .884 | 0.79 (0.47-1.34) | .390 | **1.68 (1.11-2.56)** | .015 |
| Seizure disorders | 633(1.0) | 1.08 (0.88-1.31) | .475 | 0.88 (0.71-1.11) | .284 | **1.43 (1.13-1.80)** | .002 | 1.03 (0.82-1.29) | .807 |
| Cancers | 594(.9) | 0.98 (0.77-1.26) | .899 | 1.03 (0.80-1.32) | .826 | **1.57 (1.22-2.02)** | .001 | 0.88 (0.65-1.18) | .381 |
| High cholesterol | 1807(2.8) | **1.21 (1.06-1.38)** | .006 | **1.39 (1.21-1.60)** | <.001 | **1.66 (1.43-1.92)** | <.001 | 1.14 (0.98-1.34) | .099 |
| HIV/AIDS | 83(.1) | 0.98 (0.56-1.72) | .954 | 1.49 (0.85-2.63) | .162 | 0.83 (0.39-1.76) | .624 | **2.04 (1.22-3.43)** | .007 |
| Other autoimmune disorder | 1735(2.7) | 1.13 (1.00-1.27) | .053 | **1.30 (1.15-1.47)** | <.001 | 1.10 (0.95-1.28) | .191 | 0.86 (0.74-1.00) | .054 |
| Other chronic disease | 3193(4.9) | **1.37 (1.25-1.49)** | <.001 | **1.24 (1.13-1.36)** | <.001 | **1.22 (1.10-1.36)** | <.001 | **1.48 (1.34-1.63)** | <.001 |
| **History of diagnosed mental disorder** | 23275 (35.6) | **2.75 (2.63-2.87)** | <.001 | **2.86 (2.73-3.00)** | <.001 | **1.88 (1.79-1.99)** | <.001 | **3.39 (3.21-3.57)** | <.001 |
| **COVID-19 history (testing uptake)** |  |  |  |  |  |  |  |  |  |
| Confirmed by testing (group 1) | 7158(11.0) | 1 (reference) | NA | 1 (reference) | NA | 1 (reference) | NA | 1 (reference) | NA |
| Physician diagnosed COVID-19 without testing (group 2) | 2497(3.8) | **1.42 (1.27-1.59)** | <.001 | **1.43 (1.27-1.61)** | <.001 | **1.16 (1.01-1.32)** | .030 | **1.15 (1.01-1.32)** | .036 |
| Experienced symptoms consistent with COVID-19 without testing (group 3) | 8214(12.6) | **1.33 (1.23-1.45)** | <.001 | **1.24 (1.14-1.35)** | <.001 | **1.13 (1.03-1.25)** | .013 | **1.33 (1.21-1.46)** | <.001 |
| No symptoms (group 4) | 47492(72.7) | 0.99 (0.92-1.05) | .687 | **0.89 (0.83-0.95)** | .001 | **0.85 (0.78-0.92)** | <.001 | 1.03 (0.95-1.12) | .468 |
| *Note.* aOR=adjusted odds ratio. NA=not applicable. Bold font indicates statistical significance. Sample weights were used to adjust non-response based on institutional data on sex, race/ethnicity, academic level, and grade point average. | | | | | | | | | |
